# Clinical Outcomes With Once‐Weekly Insulin Icodec Versus Once‐Daily Insulin Glargine U100 in Insulin‐Naïve and Previously Insulin‐Treated Individuals With Type 2 Diabetes: A Meta‐Analysis of Randomised Controlled Trials

**DOI:** 10.1002/edm2.480

**Published:** 2024-04-24

**Authors:** Mushood Ahmed, Aimen Shafiq, Hira Javaid, Hritvik Jain, Abdulqadir J. Nashwan, Qura Tul‐Ain, Jawad Basit

**Affiliations:** ^1^ Department of Medicine Rawalpindi Medical University Rawalpindi Pakistan; ^2^ Department of Medicine Dow University of Health Sciences Karachi Pakistan; ^3^ Department of Medicine Allama Iqbal Medical College Lahore Pakistan; ^4^ Department of Internal Medicine All India Institute of Medical Sciences (AIIMS) Jodhpur India; ^5^ Department of Nursing Hamad Medical Corporation Doha Qatar; ^6^ Department of Pharmacology Shifa Tameer‐e‐Millat University Islamabad Pakistan

**Keywords:** insulin, insulin glargine, insulin icodec, type 2 diabetes

## Abstract

**Aims:**

The once‐weekly insulin icodec, a new basal insulin analog, may positively support a reduction in injection frequency and improve adherence to therapy in type 2 diabetes (T2D). This study aimed to evaluate the safety and efficacy of insulin icodec compared with those of once‐daily glargine U100.

**Methods:**

A comprehensive literature search was conducted using PubMed/MEDLINE, Embase and the Cochrane Library from inception till September 2023. Data about clinical outcomes in both groups were extracted. Forest plots were generated using the random‐effects model by pooling odds ratios (ORs) and mean differences (MDs).

**Results:**

Five randomised controlled trials and 2019 individuals with T2DM were included. In the pooled analysis, time in range was significantly higher (MD = 4.35; 95% CI: 1.65 to 7.05; *p* = 0.002) in the icodec group than in the once‐daily glargine group. The HbA1c levels were significantly reduced (MD = −0.13; 95% CI: −0.24 to −0.03; *p* = 0.02) in the weekly icodec group compared with those in the once‐daily glargine group. The weight gain was significantly less in the glargine group than in the weekly icodec group (MD = 0.41; 95% CI: 0.04 to 0.78; *p* = 0.03); however, in the subgroup analysis, this change became statistically insignificant in both insulin‐naïve and previously insulin‐treated individuals. The results were comparable across two groups for fasting plasma glucose levels, hypoglycaemia alert (Level 1), clinically significant (Level 2) or severe hypoglycaemia (Level 3), and adverse events.

**Conclusion:**

Insulin icodec was associated with a reduction in glycated haemoglobin levels and higher time in range, with a similar safety profile as compared to insulin glargine U100. However, further evidence is still needed to reach a definitive conclusion.

## Graphical abstract

The once‐weekly insulin icodec, a new basal insulin analog, may positively support a reduction in injection frequency and improve adherence to therapy in type 2 diabetes (T2D) patients. We performed a meta‐analysis to evaluate the safety and efficacy of insulin icodec compared with those of once‐daily glargine U100.

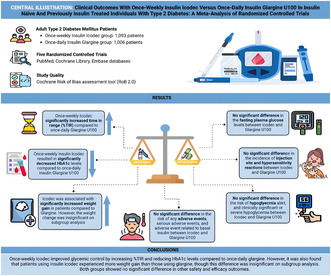

## 1 Introduction

Diabetes is a persistent and progressing disease that necessitates several interventional methods to help mitigate its detrimental effects [1]. The degenerative nature of this disease requires pharmaceutical treatment optimisation, which frequently necessitates the administration of exogenous insulin, the mainstay in treating diabetes [2]. Although many people with type 2 diabetes (T2D) get prescribed insulin therapy, glycaemic control is frequently insufficient in these individuals [3]. This poor glycaemic control is attributed to decreased adherence, which can be difficult owing to the requirement of numerous daily subcutaneous administrations, complex dosing and the potential risk of hypoglycaemia [4]. Some of these challenges have been addressed by once‐daily basal insulin analogs [5, 6], but research indicates that individuals with T2D might benefit from a further decrease in the frequency of injections. Hence, reducing the insulin therapy to once‐weekly administration may enhance adherence, improve quality of life, lower the burden of T2D and improve the glycaemic control [7].

Insulin icodec is a novel once‐weekly basal insulin analog that binds albumin robustly yet reversibly. The production of a largely inactive albumin‐bound depot, which gradually releases the active insulin icodec over time, is the primary cause of its protraction [8]. According to pharmacokinetic and pharmacodynamic studies, the glucose‐lowering impact of insulin icodec is uniformly distributed throughout a 1‐week dose interval [9]. Although a prior meta‐analysis was performed to compare the weekly use of insulin icodec with the daily use of insulin glargine regarding safety and effectiveness [10], its limited sample size and the inclusion of only Phase 2 trials make it impractical to establish any firm conclusions. To address this literature gap and to assist with improved decision‐making when choosing between weekly use of insulin icodec and daily use of insulin glargine, we conducted systematic review and meta‐analysis with a considerably larger sample size by including recently published phase 3 trials.

## 2 Materials and Methods

Our systematic review and meta‐analysis adhered to the guidelines established by the Preferred Reporting Items for Systematic Review and Meta‐Analysis (PRISMA) [11]. The PRISMA checklist is provided as Table S1.

### 2.1 Data Sources and Search Strategy

Two reviewers (M.A. and H.J.) conducted a comprehensive search on PubMed/MEDLINE, Embase and the Cochrane Library, without language restrictions, from inception until September 2023. The references of included articles were manually screened by reviewers to ensure the inclusion of all potential studies. The search methodology employed for the literature search consisted of the following keywords and related Medical Subject Headings (MeSH) terms: (((insulin icodec) OR (insulin glargine) OR (weekly icodec) OR (daily glargine) OR (insulin, long‐acting) OR (glargine U100) OR (icodec)) AND ((type 2 diabetes mellitus) OR (diabetes mellitus, type II) OR (diabetes mellitus, adult‐onset))).

### 2.2 Eligibility Criteria and Outcomes Assessed

The studies met the inclusion criteria for our systematic review and meta‐analysis if they: (a) were randomised controlled trials (RCTs), either double‐blinded or open‐labelled; (b) included adult individuals (≥18 years) with T2D mellitus individuals using oral hypoglycaemic drugs; (c) compared once‐weekly insulin icodec to once‐daily insulin glargine U100; (d) randomised at least 100 individuals; (e) had a follow‐up duration of a minimum of 8 weeks; and (f) evaluated at least one of the outcomes of interest. Our analysis excluded studies with insufficient data, case reports, case series, letters, editorials and reviews. The primary outcomes were time in range (%), change in HbA1c levels (%) and change in body weight.

The secondary outcomes included hypoglycaemia alert (Level 1), clinically significant (Level 2) or severe hypoglycaemia (Level 3), fasting plasma glucose levels, any adverse outcome, serious adverse event, any adverse event related to basal insulin, injection site reaction and hypersensitivity events.

Hypoglycaemia alert (Level 1) is defined as blood glucose levels of <70 mg/dL (<3.9 mmol/L) or ≥ 54 mg/dL (3.0 mmol/L). Clinically significant (Level 2) hypoglycaemia is defined as blood glucose levels of <54 mg/dL (3.0 mmol/L) and severe hypoglycaemia (Level 3) as an event characterised by altered mental and/or physical status requiring assistance for the treatment of hypoglycaemia [12].

### 2.3 Study Selection, Data Extraction and Quality Assessment

The search citations obtained from the literature search were transferred to the EndNote X9 Reference Manager (Clarivate Analytics, Philadelphia, USA) to remove any duplicates present. The process for selecting trials involved a thorough analysis of titles and abstracts by two independent reviewers (A.S. and M.A.), who then proceeded to examine the full texts of the articles that fulfilled the eligibility criteria for inclusion. A third reviewer (H.J.) was consulted in case of any discrepancy. The extracted data for each study included the year and design of the study, follow‐up duration and the baseline characteristics of both the intervention and control groups, including the number of participants, mean age, gender, duration of diabetes, body mass index, HbA1c levels (%), fasting plasma glucose (mg/dL), any complications of diabetes present and outcomes of interest. The RCTs were evaluated for their risk of bias using Version 2 of the Cochrane Risk of Bias assessment tool [13]. The tool assessed the risk of biases in five domains: randomisation, deviations from intended variation, missing outcome data, measurement of outcome and selection of reported results. The trials were scored based on a high, some concerns, or low risk of bias in each domain.

### 2.4 Statistical Analysis

Meta‐analysis for the extracted data was performed using the Review Manager (RevMan) software, version 5.4.1 (Nordic Cochrane Centre, Cochrane Collaboration, Denmark). For outcomes with continuous data, the mean difference (MD) values with 95% CIs were examined, while for outcomes with dichotomous data, the odds ratio (OR) values with 95% confidence intervals (CIs) were evaluated. To account for variability among the studies included, the weighted DerSimonian and Laird random‐effects model was used to pool the data [14]. Subgroup analyses were conducted for the insulin‐naïve and previously insulin‐treated individuals for the primary outcomes. A visual inspection of funnel plots was performed to evaluate the risk of publication bias. Heterogeneity was assessed using the chi‐squared test, where significant heterogeneity was indicated by *p* < 0.1. The Higgins *I* ^2^ test was also used to evaluate heterogeneity across the trials. On the scale used, low risk was indicated by values <25%, moderate risk by 25%–75% and high risk by >75% [15]. In all instances, a *p*‐value of <0.05 was considered significant.

## 3 Results

The systematic search by authors yielded 97 records. Thirty‐two duplicated studies were removed, and 42 were excluded after title and abstract screening. Full text of 23 studies was reviewed to assess their eligibility, and five studies met the predefined inclusion criteria. The detailed study selection and screening process are shown in the PRISMA flowchart in Figure S1.

### 3.1 Study Characteristics

We included five RCTs in our meta‐analysis [16, 17, 18, 19, 20]. 2019 individuals with T2DM were included, 1093 in the icodec group and 1006 in the glargine group. The studies were published from 2020 to 2023. Male individuals prevailed in all included studies. Previous basal insulin therapy was reported in two trials [18, 19], and three trials enrolled insulin‐naïve individuals [16, 17, 20]. The mean age of the participants ranged from 59.1 to 62.4 years, and the mean follow‐up duration was 32.4 weeks. The detailed characteristics of the included studies and participants are given in Table 1.

**TABLE 1:** Baseline characteristics of the included trials.

Included studies	Population	Intervention	Control	Study design	Follow‐up	HbA1c for inclusion	Target fasting glucose (mg/dL)	Previous basal insulin therapy	No. of individuals, *n*	Male sex *n* (%)	Age (mean ± SD)	Diabetes duration (years) (mean ± SD)	BMI (mean ± SD)	HbA1c (mean ± SD)	Fasting plasma glucose levels (mean ± SD)
Rosenstock, 2020	T2DM on OHA	Once‐weekly icodec	Once‐daily glargine U100	RCT	26 weeks +5	7.0%–9.5%	70–108	No	IV = 125 C = 122 Total = 247	IV = 70 (56.0%) C = 69 (56.6%)	IV = 59.7 ± 8.2 C = 59.4 ± 9.5	IV = 10.5 ± 8.4 C = 8.8 ± 6.1	IV = 31.1 ± 4.9 C = 31.4 ± 4.4	IV = 8.09 ± 0.70 C = 7.96 ± 0.65	IV = 182 ± 42 C = 180 ± 42
Bajaj, 2021	T2DM on OHA	Once‐weekly icodec with loading dose	Once‐daily glargine U100	RCT	16 weeks +5	7%–10%	70–130	Yes	IV = 54 C = 50 Total = 104	IV = 39 (72.2%) C = 33 (66.0%)	IV = 62.4 ± 7.2 C = 60.5 ± 7.9	IV = 13.8 ± 7.7 C = 14.8 ± 8.1	IV = 30.2 ± 4.3 C = 30.3 ± 5.0	IV = 7.8 ± 0.7 C = 7.9 ± 0.7	IV = 142 ± 34 C = 148 ± 36
Lingvay, 2021	T2DM on OHA	Once‐weekly icodec titration B	Once‐daily glargine U100	RCT	16 weeks +5	7%–10%	80–130	No	IV = 51 C = 51 Total = 102	IV = 54.9% C = 52.9%	IV = 61.2 ± 8.0 C = 60.2 ± 8.1	IV = 9.6 ± 4.9 C = 11.8 ± 6.8	IV = 31.4 ± 4.7 C = 30.6 ± 4.7	IV = 8.1 ± 0.8 C = 8.2 ± 0.8	IV = 180 ± 38 C = 168 ± 42
Mathieu, 2023	T2DM on OHA	Once‐weekly icodec	Once‐daily glargine U100	RCT	26 weeks +5	7%–10%	80–130	Yes	IV = 291 C = 291 Total = 582	IV = 154 (53%) C = 150 (52%)	IV = 59.7 ± 10.1 C = 59.9 ± 9.9	IV = 18.0 ± 9.1 C = 16.3 ± 7.7	IV = 30.5 ± 5.0 C = 30.0 ± 5.0	IV = 8.29 ± 0.86 C = 8.31 ± 0.90	IV = 167 ± 54 C = 173 ± 63
Rosenstock, 2023	T2DM on OHA	Once‐weekly icodec	Once‐daily glargine U100	RCT	78 weeks +5	7%–11%	70–180	No	*N* = 492 *N* = 492 *N* = 984	IV = 295 (60.0%) C = 263 (53.5%)	IV = 59.1 ± 10.1 C = 58.9 ± 9.9	IV = 11.6 ± 6.7 C = 11.5 ± 6.8	IV = 30.0 ± 4.8 C = 30.1 ± 5.1	IV = 8.5 ± 1.0 C = 8.4 ± 1.0	IV = 185.3 ± 49.0 C = 185.7 ± 51.7

Abbreviations: BMI, body mass index; C, control; HbA1C, glycated haemoglobin; IV, intervention; OHA, oral hypoglycaemic agents; RCT, randomised controlled trial; T2DM, type 2 diabetes mellitus.

### 3.2 Quality Assessment of Included Studies

Four studies were of high methodological quality. However, some concerns of bias were reported in one trial. The detailed bias assessment for each included trial is provided as Figure S2. The funnel plots for each outcome are provided as Figures S3–S12.

### 3.3 Results of the Meta‐Analysis

#### 3.3.1 Time in Range (%)

**FIGURE 1:**
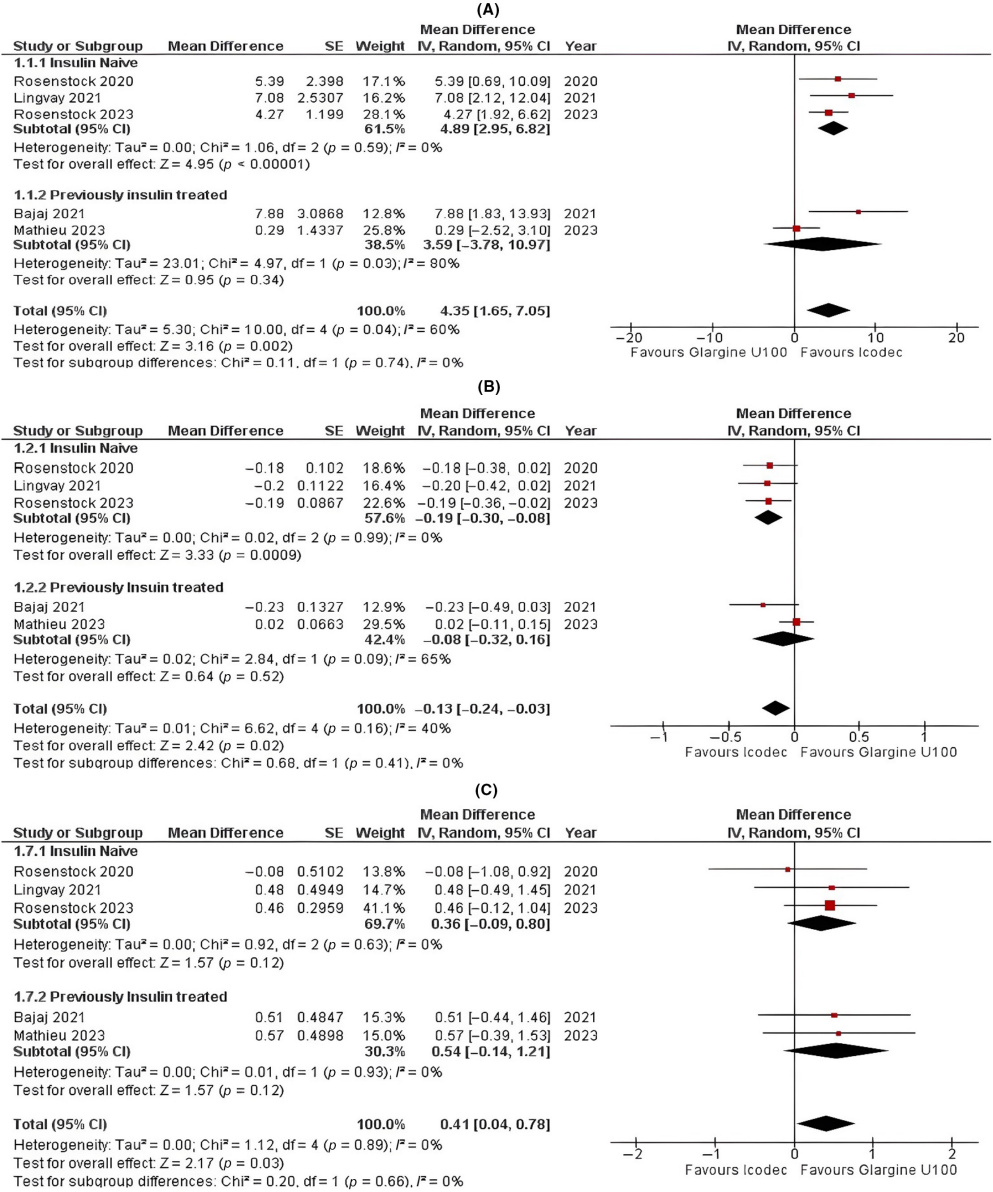
Forest plots of once‐weekly insulin icodec versus once‐daily insulin glargine U100 for (A) time in range with glucose, (B) change in HbA1c and (C) change in body weight.

Our pooled analysis showed that once‐weekly icodec was associated with a significantly higher time in range (% TIR) than once‐daily glargine U100 (MD = 4.35; 95% CI: 1.65 to 7.05; *p* = 0.002; *I* ^2^ = 60%; Figure 1A). The exclusive analysis of the three studies with insulin‐naïve individuals and two studies with previously insulin‐treated individuals demonstrated that insulin‐naïve individuals who received once‐weekly icodec had a significantly higher %TIR (MD = 4.89; 95% CI: 2.95 to 6.82; *p* < 0.00001; *I* ^2^ = 0%). However, for previously insulin‐treated individuals, there was no statistically significant difference between insulin icodec and glargine (MD = 3.59; 95% CI: −3.78–10.97; *p* = 0.34; *I* ^2^ = 80%). The difference between the insulin‐naïve and previously insulin‐treated subgroups was not statistically significant (*p* = 0.74).

#### 3.3.2 Change in HbA1c (%)

In our pooled analysis, once‐weekly icodec resulted in a significant decrease in HbA1c levels compared with once‐daily glargine U100 (MD = −0.13; 95% CI: −0.24 to −0.03; *p* = 0.02; *I* ^2^ = 40%; Figure 1B). In an exclusive analysis, the insulin‐naïve patient group showed a statistically significant reduction in HbA1c levels (MD = −0.19; 95% CI: −0.30 to −0.08; *p* = 0.0009; *I* ^2^ = 0%); however, no statistically significant difference was observed in HbA1c levels in the icodec and glargine U100 groups for previously insulin‐treated individuals (MD = −0.08; 95% CI: −0.32 to 0.16; *p* = 0.52; *I* ^2^ = 65%). No statistically significant difference was observed between the two subgroups (*p* = 0.41).

#### 3.3.3 Change in Body Weight (kg)

The change in body weight was found to be statistically significant with individuals gaining less weight who received once‐daily glargine than the individuals who received once‐weekly icodec (MD = 0.41; 95% CI: 0.04 to 0.78; *p* = 0.03; *I* ^2^ = 0%; Figure 1C). The exclusive analysis showed no statistically significant difference in the icodec and glargine groups for both insulin‐naïve individuals (MD = 0.36; 95% CI: −0.09 to 0.80; *p* = 0.12; *I* ^2^ = 0%) and those who were previously treated with insulin (MD = 0.54; 95% CI: −0.14 to 1.21; *p* = 0.12; *I* ^2^ = 0%). The two subgroups had no statistically significant difference (*p* = 0.66).

#### 3.3.4 Fasting Plasma Glucose Levels (mg/dL)

**FIGURE 2:**
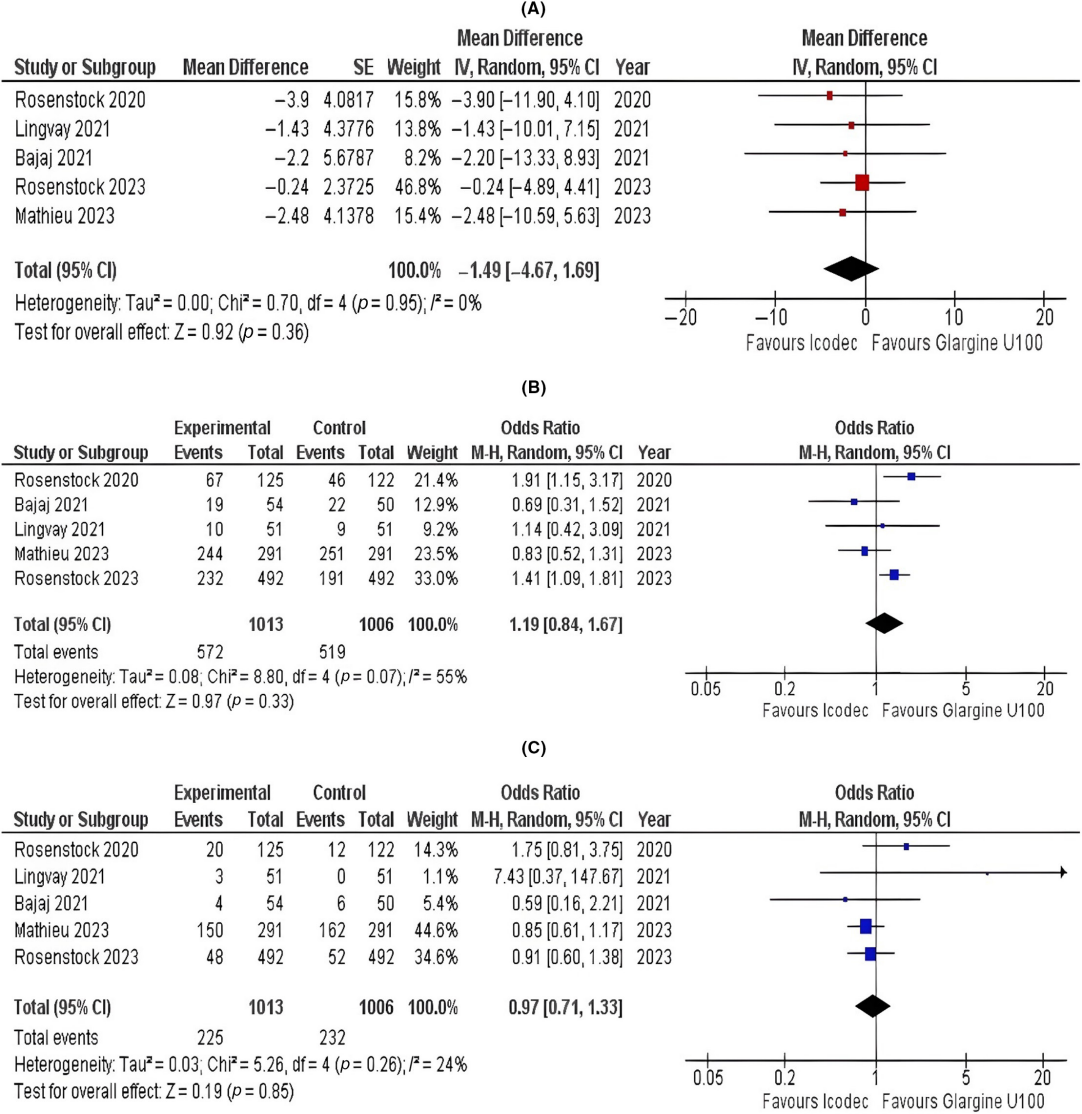
Forest plots of once‐weekly insulin icodec versus once‐daily insulin glargine U100 for (A) fasting plasma glucose levels, (B) hypoglycaemia alert (Level 1) and (C) clinically significant (Level 2) or severe hypoglycaemia (Level 3).

Our pooled analysis did not show a statistically significant difference in the fasting plasma glucose levels (mg/dL) between individuals who received either icodec or glargine U100 (MD = −1.49; 95% CI: −4.67 to 1.69; *p* = 0.36; *I* ^2^ = 0%; Figure 2A).

#### 3.3.5 Hypoglycaemia

There was no statistically significant difference in the risk of hypoglycaemia alert (Level 1) in individuals who received once‐weekly icodec and those who received once‐daily glargine U100 (OR = 1.19; 95% CI: 0.84 to 1.67; *p* = 0.33; *I* ^2^ = 55%; Figure 2B). The pooled analysis showed no statistically significant difference for clinically significant (Level 2) or severe hypoglycaemia (Level 3) in the icodec group compared with the glargine U100 group (OR = 0.97; 95% CI: 0.71 to 1.33; *p* = 0.85; *I* ^2^ = 24%; Figure 2C).

#### 3.3.6 Adverse Events

**FIGURE 3:**
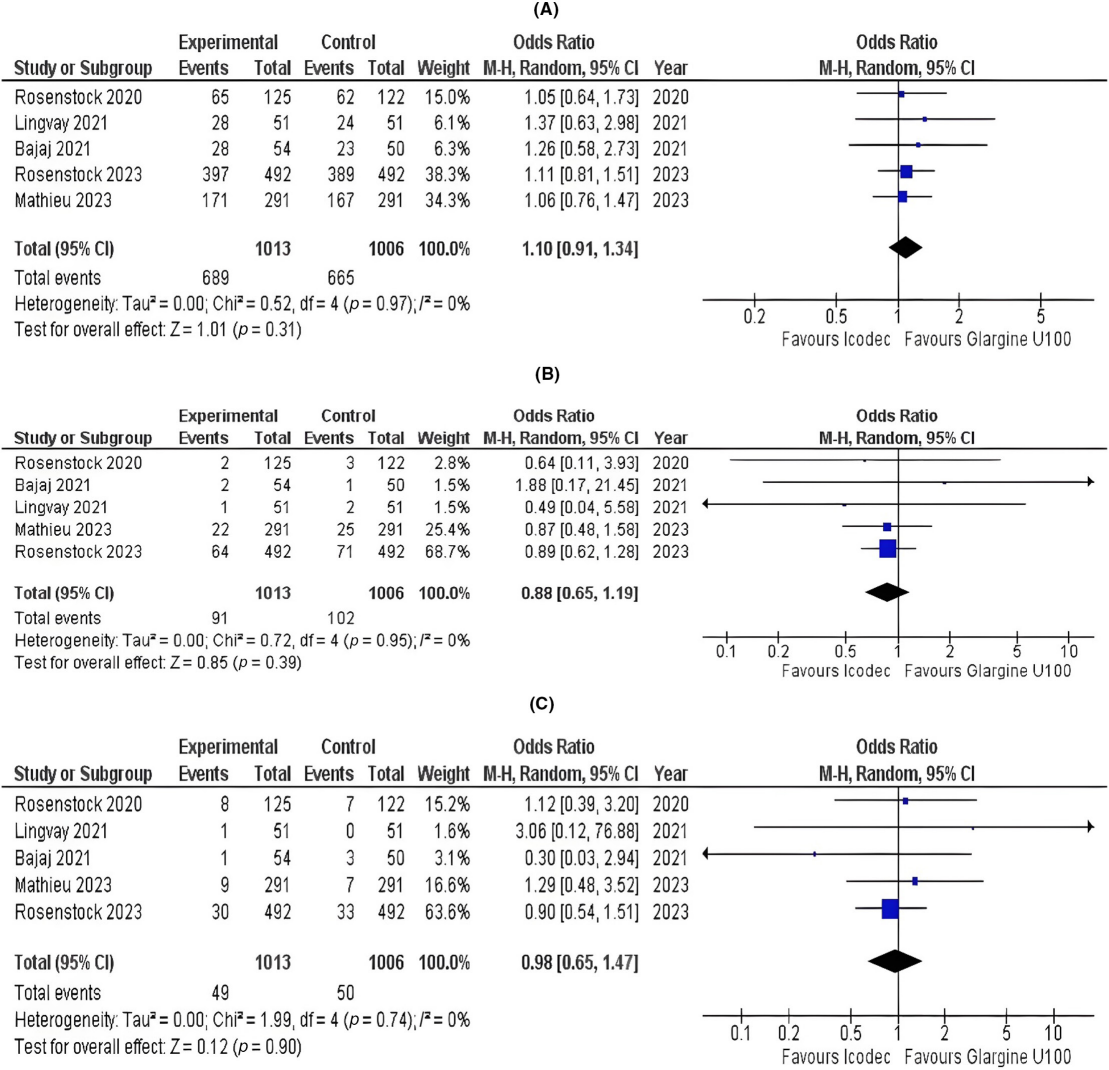
Forest plots of once‐weekly insulin icodec versus once‐daily insulin glargine U100 for (A) risk of any adverse event, (B) risk of serious adverse events and (C) risk of any adverse event related to basal insulin.

The risk of any adverse event was comparable across the two groups (OR = 1.10; 95% CI: 0.91 to 1.34; *p* = 0.31; *I* ^2^ = 0%; Figure 3A). The pooled analysis showed that the risk of serious adverse events had no statistically significant difference in the group taking icodec compared with the group taking glargine U100 (OR = 0.88; 95% CI: 0.65 to 1.19; *p* = 0.39; *I* ^2^ = 0%; Figure 3B). No statistically significant difference for any adverse event related to basal insulin was observed (OR = 0.81; 95% CI: 0.65 to 1.47; *p* = 0.90; *I* ^2^ = 0%; Figure 3C).

#### 3.3.7 Injection Site Reaction and Hypersensitivity Reactions

**FIGURE 4:**
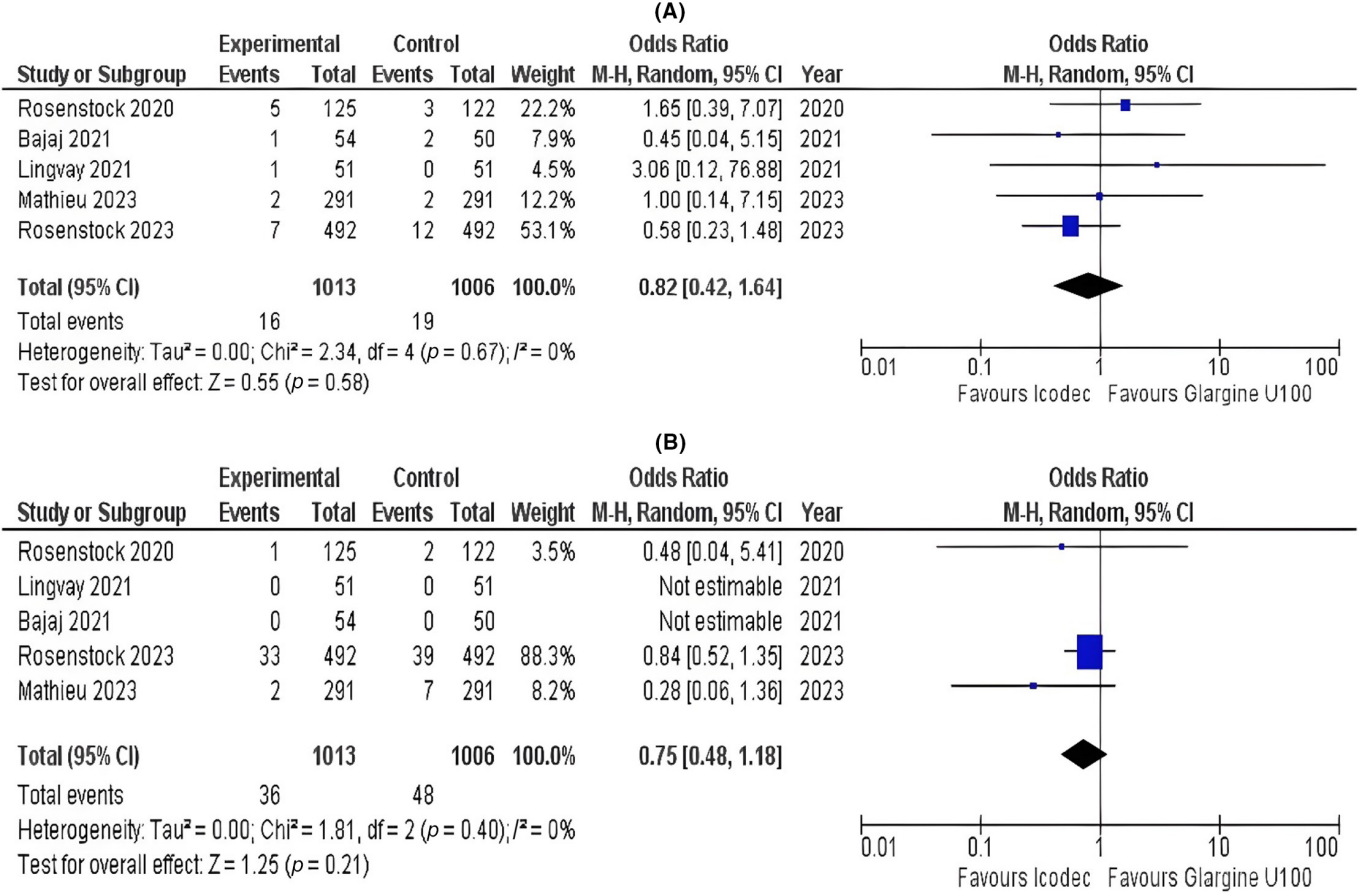
Forest plots of once‐weekly insulin icodec versus once‐daily insulin glargine U100 for (A) injection site reactions and (B) hypersensitivity reactions.

Injection site reactions did not show statistically significant difference between once‐weekly icodec and once‐daily glargine (OR = 0.82; 95% CI: 0.42 to 1.64; *p* = 0.58; *I* ^2^ = 0%; Figure 4A). Our pooled analysis demonstrated that the hypersensitivity reactions had no statistically significant difference in the once‐weekly icodec group compared with the once‐daily glargine group (OR = 0.75; 95% CI: 0.48 to 1.18; *p* = 0.21; *I* ^2^ = 0%; Figure 4B).

## 4 Discussion

In this systematic review and meta‐analysis of 5 RCTs and 2019 individuals, we sought to compare the safety and efficacy of once‐weekly insulin icodec with once‐daily insulin glargine U100 in individuals with T2D. Our pooled analysis demonstrated a statistically higher %TIR and significantly reduced HbA1c in the Icodec group compared with the glargine group. The change in body weight was statistically significant, with individuals gaining less weight in the glargine group than those who received once‐weekly icodec. However, no significant difference was observed between the two groups regarding fasting plasma glucose levels, hypoglycaemia alert (Level 1), clinically significant (Level 2) or severe hypoglycaemia (Level 3), any serious adverse events, adverse events related to basal insulin levels, injection site reaction and hypersensitivity reaction.

The requirement of fewer injections with once‐weekly icodec instead of a once‐daily glargine may enhance patient adherence, resulting in better glycaemic control in individuals with T2D [7]. Hence, the comparison of safety and clinical endpoints between the two is essential. Although a previous meta‐analysis [10] has been conducted on this topic, it lacked the inclusion of any Phase 3 trials. Our meta‐analysis addressed this literature gap by including large multicentre, Phase 3 trials. Hence, in contrast to the previous meta‐analysis, we reached statistically significant results with individuals gaining less weight who received once‐daily glargine. But on performing subgroup analysis, weight gain was insignificant in both insulin‐naïve and previously insulin‐treated individuals. The possibility that once‐weekly icodec could prove to be a more efficient choice when commencing insulin therapy is supported by the results of this meta‐analysis that demonstrated significantly superior outcomes of %TIR and change in HbA1c from baseline in the icodec group while having a similar safety profile to the glargine group. Additionally, a once‐weekly option might facilitate treatment initiation in T2D individuals who have not previously taken insulin by minimising clinical inertia and promoting higher acceptance of insulin therapy [16].

The International Consensus recommends a mean TIR of >70% [21]. Additionally, it is well established that each 5% increase in TIR is linked to clinically significant benefits. One study evaluating 1440 individuals demonstrated that a 10% decrease in TIR raised the hazard ratio of microalbuminuria by 40% and retinopathy by 64% (*p* ≤ 0.001) [22]. Hence, our pooled analysis, resulting in a significant rise in TIR in the icodec group compared with the glargine group, indicates that the use of once‐weekly icodec can contribute to a possible decrease in the complications of diabetes. Furthermore, no statistically significant difference was observed between once‐weekly icodec and once‐daily glargine U100 for adverse events. However, in a recently published ONWARDS 6 trial [23] that compared safety and efficacy of once‐weekly icodec with once‐daily degludec in type 1 diabetes, the use of icodec was associated with a statistically significantly higher combined Level 2 or Level 3 hypoglycaemia (baseline to Week 26). The use of icodec was also associated with a range of adverse events that included infections, diabetic retinopathy, disorders of the nervous system, pyrexia and arthralgia [23]. Similar findings were observed in a meta‐analysis that compared once‐weekly icodec with daily basal insulin analogs [24]. These findings warrant the need for further evidence and large multicentre RCTs to establish the safety and efficacy of once‐weekly icodec.

It is important to address that despite achieving statistically significant results for change in HbA1c from baseline in the icodec group compared with the once‐daily glargine group across our included studies, the real‐world data have failed to achieve clinical significance. Hence, more real‐world studies are needed to articulate a definitive conclusion.

The required insulin icodec dosage can differ across different populations. This can be attributed to variations in insulin secretion capacity in different racial groups. For example, the insulin secretion capacity in Asian individuals with T2D is different from Caucasian populations [25]. These variations can have a clinically profound impact as they have the potential to influence the required dosage of insulin icodec.

Furthermore, the Lingvay study compared three alternative icodec titration algorithms based on prebreakfast self‐measured glucose goals and weekly insulin dosage adjustments [20]. Since icodec titration B employed a 28 unit/week adjustment and a prebreakfast goal of 80–130 mg/dL that is in accordance with ADA guidelines, we included this group in our analysis [26]. Additionally, titration A employed a 21 unit/week adjustment, which was different from Rosenstock et al. [16] and Bajaj [18] studies, and titration C used a different goal than the glargine U100 group, which could have introduced bias in the results. Furthermore, the Bajaj trial [17] also evaluated two icodec treatment plans, one with and one without an initial loading dosage (LD). The discontinuation of prior basal insulin might cause glycaemic control to worsen since insulin icodec has a half‐life of 1 week and reaches its steady state after 3–4 weeks [18, 20]. Consequently, an initial loading dose could mitigate the effects of this transition. Hence, we decided to include the LD group in this meta‐analysis.

Additionally, all the included studies were conducted based on whether the individuals were insulin‐naïve or previously insulin‐treated. As a result, if a patient has already received treatment, their response to insulin therapy could differ, which could have impacted our findings. Hence, we performed a subgroup analysis of studies with insulin‐naïve or previously insulin‐treated individuals for the primary outcomes to overcome this.

Although a previous meta‐analysis has been performed on this topic, including only Phase 2 trials makes it impractical to establish any firm conclusions. To the best of our knowledge, this is the first systematic review and meta‐analysis that compared the safety and efficacy of once‐daily glargine with once‐weekly icodec in T2D individuals with data pooled from multicentre Phase 3 clinical trials. Additionally, we performed a subgroup analysis to accurately evaluate the impact of starting insulin therapy with glargine and icodec on insulin‐naïve or previously insulin‐treated individuals. Thus, the current state of the literature on this topic and the total impact magnitude are accurately evaluated.

This study has some limitations. Firstly, this is a study‐level meta‐analysis, and the absence of patient‐level information made it challenging to address individual confounding. Secondly, significant heterogeneity was observed in some of the outcomes, which could be attributed to the variation in the length of the follow‐up period among the included studies, the evaluation of two distinct icodec treatment plans in one study and the inclusion of three alternative icodec titration algorithms in another study. Moreover, the data regarding safety and efficacy of once‐weekly icodec in previously insulin‐treated patients are very limited. This shows the need for more clinical studies to evaluate the impact of icodec previously insulin‐treated patients. There is also a difference in fasting glucose levels and target levels in the included studies that could have impacted the findings.

In conclusion, once‐weekly insulin icodec resulted in a higher %TIR and a reduction in glycated haemoglobin levels compared with once‐daily glargine U100. Individuals who received once‐daily glargine gained less weight, but in subgroup analysis, this change was comparable across both treatment arms. The safety profile of both insulin forms is similar. However, results should be interpreted cautiously as the data regarding the safety and efficacy of once‐weekly icodec are very limited. Hence, more Phase 3 trials with large sample sizes are required to reach a definitive conclusion.

## Author Contributions

**Mushood Ahmed:** Conceptualization (lead); Data curation (lead); Formal analysis (lead); Methodology (lead); Supervision (lead); Visualization (lead); Writing – Original draft (lead); Writing – Review and editing (lead). **Aimen Shafiq:** Supervision (equal); Visualization (equal); Writing – Original draft (equal). **Hira Javaid:** Formal analysis (equal); Methodology (equal); Software (equal); Writing – Original draft (equal). **Hritvik Jain:** Formal analysis (equal); Methodology (equal); Visualization (equal); Writing – Original draft (equal). **Abdulqadir J. Nashwan:** Conceptualization (equal); Supervision (equal); Writing – Review and editing (equal). **Qura Tul‐Ain:** Formal analysis (equal); Methodology (equal); Visualization (equal); Writing – Original draft (equal). **Jawad Basit:** Formal analysis (equal); Methodology (equal); Visualization (equal); Writing – Original draft (equal).

## Conflicts of Interest

The authors declare no conflicts of interest.

## Supporting information


Appendix S1

## Data Availability

Data will be available upon request.
